# Forest dynamics and its driving forces of sub-tropical forest in South China

**DOI:** 10.1038/srep22561

**Published:** 2016-03-04

**Authors:** Lei Ma, Juyu Lian, Guojun Lin, Honglin Cao, Zhongliang Huang, Dongsheng Guan

**Affiliations:** 1 School of Environmental Science and Engineering, Sun Yat-sen University, No. 135, Xingang Xi Road, Guangzhou, 510275, P.R. China; 2 Guangdong Provincial Key Laboratory of Environmental Pollution Control and Remediation Technology, Sun Yat-sen University, No. 135, Xingang Xi Road, Guangzhou, 510275, P.R. China; 3 Key Laboratory of Vegetation Restoration and Management of Degraded Ecosystems, South China Botanical Garden, Chinese Academy of Sciences, Xingke Road 723, Guangzhou, 510650, P.R. China; 4 Changjiang Water Resources Protection Institute, Qintai Road 515, Wuhan, 430051, P.R. China

## Abstract

Tree mortality and recruitment are key factors influencing forest dynamics, but the driving mechanisms of these processes remain unclear. To better understand these driving mechanisms, we studied forest dynamics over a 5-year period in a 20-ha sub-tropical forest in the Dinghushan Nature Reserve, South China. The goal was to identify determinants of tree mortality/recruitment at the local scale using neighborhood analyses on some locally dominant tree species. Results show that the study plot was more dynamic than some temperate and tropical forests in a comparison to large, long-term forest dynamics plots. Over the 5-year period, mortality rates ranged from 1.67 to 12.33% per year while recruitment rates ranged from 0 to 20.26% per year. Tree size had the most consistent effect on mortality across species. Recruitment into the ≥1-cm size class consistently occurred where local con-specific density was high. This suggests that recruitment may be limited by seed dispersal. Hetero-specific individuals also influenced recruitment significantly for some species. Canopy species had low recruitment into the ≥1-cm size class over the 5-year period. In conclusion, tree mortality and recruitment for sixteen species in this plot was likely limited by seed dispersal and density-dependence.

Forests are repositories of much of the world’s biodiversity and play a crucial role in the regulation of global climate. Forests are also under tremendous pressure from human development. Identifying and understanding changes in forest composition and structure is critical to understanding how forests are responding to environmental variability and changes^1^,^2^,^3^. Understanding the dynamics of plant community composition is fundamental to understanding numerous ecological processes^4^. The study of community dynamics in complex, species-rich ecosystems such as tropical forests is important as they are globally significant ecosystems for both biodiversity and climate^5^. Researchers^6^ have compared the dynamics of tropical forest communities from different parts of the world and found highly variable rates of recruitment, growth and mortality highlighting different mechanisms for different tropical systems. This research suggests that resilience to environmental changes will vary across different tropical ecosystems.

Mortality, growth and recruitment of tree species are key factors influencing the structure, composition and succession of forest communities^7^. Tree mortality is recognized as one of the most important processes in forest dynamics and is influenced by many factors^7^. It can facilitate turnover in species composition, affect community structure, and alter rates of nutrient cycling or biomass accumulation^8^. It can also determine forest dynamics or succession and contribute to tree species coexistence^9^. The mortality probability of an individual tree is commonly examined as a function of readily measured variables such as tree size, recent growth and the spatial pattern of surrounding trees also known as the competitive neighborhood^10^,^11^. An understanding of tree mortality is central to any predictive understanding of forest dynamics^12^.

Forest growth and recruitment are also important processes shaping forest structure and dynamics^13^. The seedling to sapling transition is a critical bottleneck in tree establishment^14^. The spatial pattern of seedling recruitment influences the long-term distribution patterns of species^15^, and can have significant effects on the composition and abundance of plant communities^16^. Theoretically, recruitment limitation has been shown to promoting species coexistence and the maintenance of community diversity^14^. Therefore, factors that influence seedling recruitment are of great importance to forest ecologists and researchers^17^.

Negative con-specific density and frequency dependence are widely recognized as prominent mechanisms of species coexistence. Several hypotheses, such as the Janzen-Connell hypothesis, consider their effects on community assembly^18^,^19^. Experimental and observational studies have found patterns of distance- and density-dependent seedling recruitment and mortality of tree species consistent with the Janzen-Connell hypothesis in both temperate and tropical communities^20^,^21^. These studies point to density dependence as an important stabilizing force promoting species coexistence in forest systems^22^. Dispersal limitation is another mechanism driving of species diversity. Dispersal limitation refers to the phenomenon of declining seed density with increasing distance from the maternal tree^7^. Seeds of superior competitors may fail to arrive at suitable micro-sites and less competitive species will have more chances to take their places, thus, slowing competitive exclusion and promoting species coexistence^23^.

In local neighborhood analyses, previous studies have indicated that the spatial auto-correlation of seedling survival was important at small spatial scales (1–5 m) but decayed rapidly with increasing distance. Negative density dependence is thought to be strongest for young seedlings, which are highly vulnerable to attack by natural enemies^10^,^11^. Although more resistant than new seedlings, established seedlings are frequently resource-limited by asymmetric competition with larger individuals^24^,^25^ and may lack adequate resources to tolerate or recover from damage. Thus, established seedlings are also likely to exhibit lower survival rates in areas of high con-specific density^26^. Previous studies have suggested that con-specific seedling density had a greater negative effect on survival than hetero-specific seedling density as well as being stronger and extending farther for rare species. Some studies indicate that negative density dependence can promote species coexistence in rain forest communities but that the scale dependence of interactions differs between rare and common species^4^,^27^.

Local neighborhood conditions broadly include two major factors, biotic and abiotic variables. The effects of biotic and abiotic variables represent two important explanations for species coexistence in ecological communities: frequency (or density) dependence and resource niche partitioning, respectively^9^,^28^. Competition has been found to be the primary factor in long-term changes in tree mortality, growth, and recruitment^29^. Regional climate has a weaker yet still significant effect on tree mortality, but little effect on tree growth and recruitment. This indicates that internal community level processes, more so than external climatic factors, are driving forest dynamics^30^.

Forest dynamics are slow-acting, and thus require long-term data to be accurately characterized^31^. Much of our current knowledge of long-term forest dynamics is derived from tropical forests, especially from large-scale and stem-mapped permanent forest dynamics plots^11^. Many of these permanent plots have logged multi-decadal demographic datasets, which are critical for understanding spatio-temporal variation in ecological processes. Demographic data, such as recruitment, growth and mortality offer keys to understanding directional changes in forest processes such as community composition^18^.

In this study, we evaluate the forest population dynamics of 195 tree species over 5 years in the Dinghushan sub-tropical forest permanent plot (DHS plot) in South China, with the goal of understanding forest dynamics, and determining the effects of neighborhood on tree mortality and growth. Data from the 20-ha DHS plot was used to examine biotic forces and the effects of con-specific neighbors on tree mortality and seedling recruitment. We attempt to determine whether the tree community and populations remain stable during the study period, as well as to identify and explain any changes observed. We used a modeling approach to answer three main questions: (1) How is forest dynamics (tree mortality and recruitment) influenced by biotic factors? (2) How does the species’ ecological status influence forest dynamics? (3) What is the role neighborhoods play in seedling recruitment?

## Results

### Floristic composition and vegetation structure

**Figure 1:**
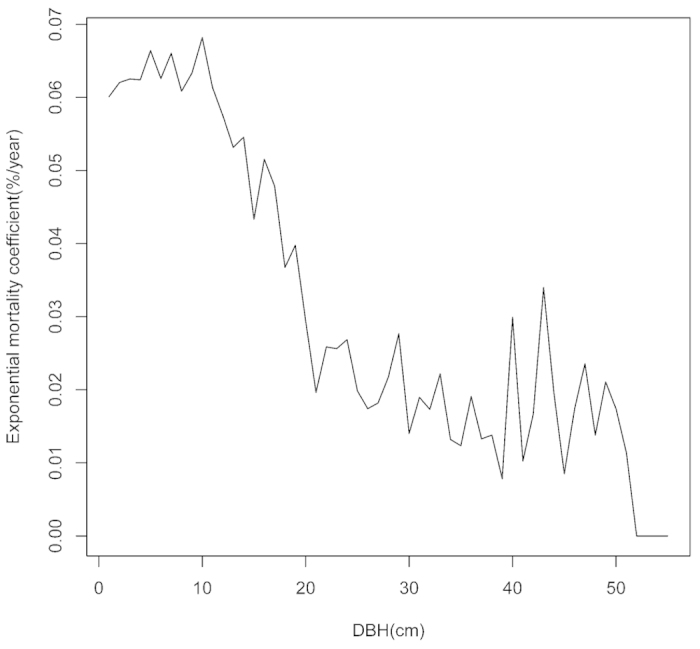
Exponential mortality coefficient decreased as the DBH of the individuals with DBH <56 cm in DHS plot increased.

During the 5-year period (2005-2010) mortality rates of the study species ranged from 1.67 to 12.33% per year while the mean recruitment rate was 3.17% per year, and ranged from 0 to 20.26% per year among species. The total number of species in the Dinghushan plot (DHS plot) decreased from 195 to 178 with 20 lost species and three new species (*Bridelia tomentosa, Michelia skinneriana, Glochidion puberum*). Average species turnover rate was 1.2% per year. The total number of stems decreased from 80504 in 2005 to 68467 in 2010, including mortality of 20424 stems (25.3% of total stems in 2005) and recruitment of 8387 stems (12.2% of total stems in 2010). The mean relative growth rate (RGR) for 101 species was 0.032 cm/y, and ranged from 0.005 to 0.1713 cm/y. The average exponential mortality coefficient was 8.01% per year, and ranged from 0 to 43.3%per year among species. In general, exponential mortality coefficient decreased as DBH increased (Fig. 1, Tables 1 and 2).

Basal area decreased from 30.1 m^2^/ha in 2005 to 26.6 m^2^/ha in 2010, resulting in a decrease of 4.16 m^2^/ha due to mortality and an increase of 0.64 m^2^/ha due to DBH growth and recruitment (0.39 m^2^/ha and 0.25 m^2^/ha, respectively). The number of stems in the plot decreased for 86 out of 101 species, resulting in a mean annual population change rate of −4.8% per year. Among the 178 tree species, the basal area of 71 species increased during the 5-year period due to either the growth of live wood (e.g. *Castanopsis chinensis*) or increment of abundance (e.g. *Aidia canthioides*). The basal area of the rest of species decreased due to tree mortality, especially for *Craibiodendron scleranthum var. kwangtungense*, *Engelhardtia roxburghiana* and *Neolitsea pallens*(Tables 1 and 2).

### Local scale drivers of tree survival

In order to detect the local drivers of tree survival in our study plot, each of the 16 species with >1400 stems were analyzed. Values of Nagelkerke’s R^2^_N_ of the best-fit models for these species were significantly different. Nagelkerke’s R^2^_N_ ranged from 0.012 (*Ormosia glaberrima*) to 0.128 (*Craibiodendron scleranthum var. kwangtungense*) (Table 3).

The main forest layer had the lowest amount of dead wood, followed by the second forest layer while the understory had the greatest number of dead individuals. The effects of tree size and biotic factors on tree survival varied among these 16 species. Tree size had the most consistent effect on mortality across species: for nine of the 16 species, tree size had the strongest positive effect on survival; for five of the 16 species, tree size had the strongest negative effect on survival. The remaining two species (*Aidia canthioides* and *Cryptocarya chinensis*) showed no effect of tree size on tree survival. Among the 16 species, 13 species were affected by biotic factors. Among the biotic factors that showed effects on tree survival, T-BA, T-N, Cons-BA, and Cons-N had significantly positive effects on tree survival for five, eight, one, and three species, respectively. Only one species (*Lindera metcalfiana*) showed a significant negative relationship with T-BA (Table 3).

### Local scale drivers of tree recruitment

The live wood of recruits of most species studied had the same trend as results simulated at all four neighborhood radii. The recruits of most of the sixteen species studied had greater observed T-BA, Cons-BA and Cons-N means than randomly generated points and most of the differences were significant (Table 4). The observed T-BA was significantly larger than random points for 10 of the 16 species at all four neighborhood radii. Fifteen of sixteen species (except for *Aidia canthioides*) had larger observed Cons-BA than simulated results. The observed T-N was significantly lower than random points for six of sixteen species, higher than random points for 5 of 16 species (Table 4, Appendix 1).

The dead wood of most recruited species studied was significantly influenced by T-BA and T-N. In addition, for most of 16 species studied the observed and randomly generated points were similar at all four neighborhood radii. The recruits of eight of the sixteen species studied had larger observed T-BA means than randomly generated points and all of the differences were significant (Table 5). The observed T-N was significantly higher than random points for ten of sixteen species, lower than random points for 5 of 16 species (Table 5, Appendix 2). In addition, the neighbors around the target tree had higher basal area and higher abundance of dead wood. In general, some recruits tended to growth in forest gaps caused by tree mortality, others were shade-tolerant species.

## Discussion

In this study, we evaluated forest dynamics and its determinants of a 20-ha permanent plot. The results of this study indicate that for the 5-year period (2005–2010) evaluated, the community dynamics of the study forest were characterized by an imbalance between mortality and recruitment rates. Species composition and community structure both were varied during this period. Mortality was greater than recruitment. Tree mortality was significantly influenced by tree size followed by biotic factors. Tree recruitment was significantly influenced by live wood and dead wood in our study plot. Overall, our results indicated that this plot was in a dynamic equilibrium status, and will remain relative stable in the future.

Ecological theory predicts that in mature ecosystems species richness, the number of individuals and the biomass of individuals will remain in a relatively stable state of equilibrium^32^. Though the total number of species in the Dinghushan 20-ha plot decreased from 195 to 178 over a 5-year period, the ecosystem showed variance in species abundance instead of species replacement. This result is consistent with previous studies^33^,^34^. This suggests demographic shifts in some species populations, which, due to low number of recruits, have trouble surviving in the community^35^.

Empirical and theoretical studies suggest that small tree size classes have higher mortality rates, although there is no consensus on the shape of the relationship between tree size and survival. In general, the probability of tree mortality decreases as trees get larger (Fig. 1), because they have the ability to withstand environmental stress. In the DHS forest plot, tree size did show a strong negative effect on tree mortality for most species, but the estimated relationship between tree mortality and size varied among tree species and forest layers^31^. Our results were consistent with results from a plot-based forest study that found that nonrandom mortality with respect to species identity occurred more often in the smaller size classes^36^. In this study plot, the main forest layer had the lowest amount of dead wood, followed by the second forest layer while the understory had the greatest number of dead individuals. This is likely because larger size trees in the main canopy are less sensitive to their neighbors due to the asymmetric nature of competition for environmental resources, such as water, nutrient and light^13^. Small Individuals had a higher mortality rate due to tree species competition. This indicates that small individuals are subject to greater competition pressure and may also be more likely to be influenced by habitat variables, since larger trees are typically found in preferred habitats due to environmental filtering. Therefore, metabolic ecology theory might not be applicable to this sub-tropical forest, as has been previously demonstrated for tropical forests^37^. A possible cause is that metabolic ecology theory assumes that different size classes receive and use the same amount of energy^38^. This assumption may be correct in even-age forests, but does not extend to this natural mixed-aged forest^29^.

All individuals in our first census in 2005 had similar diameter structure, even in different layers. In a study of an old-growth conifer forest in the USA^39^, neighborhood variables, such as all stems and con-specific density, improved models used to predict tree survival. Our results were also consistent with results from a tropical forest^40^ where they found that tree mortality was size-dependent, but also susceptible to crowding effects of neighboring trees and species life-history traits. Tree survival increases as trees become larger, but larger trees are more susceptible to human disturbance, hurricanes or lightning^41^.

We found that the subtropical forest plot in Dinghushan was much more dynamics than other tropical and temperature forest used for comparison. For example, a study of sub-tropical 25-ha plot in northeast China^42^ had similar results but overall lower dead wood, mortality rate, and recruitment rates. A 5-ha evergreen broad leaved forest in Gutianshan in northern sub-tropical China had a higher recruitment rates and lower mortality than our study plot. This may be due to the fact that the forest is at an earlier stage of succession. The pace of forest dynamics is the result of numerous factors, including topography, geology and climate, organisms present and the stage of succession, as well as anthropogenic factors in lands surrounding the reserve^35^. Of these, topography most distinguishes Dinghushan plot from the other forest dynamics plots. The elevation of Dinghushan plot varies from 230 m to 470 m and contains numerous extremely steep slopes^43^. Topography is considerably more complicated than in the other forest plots. We suggest that greater topographic variation in the Dinghushan plot may contribute to greater demographic dynamics than the other forest dynamics plots.

There is debate on the relative importance of abiotic and biotic variables on tree survival and species coexistence in forest communities, but researchers generally agree that these mechanisms are not mutually exclusive^42^,^44^. Model results show that Cons-N and Cons-BA tended to have a significant positive effect on tree survival, which indicating that neighbors of the same species have an stronger effects than neighbors of different species, likely due to strong inter-specific competition or natural enemy effects. We propose that con-specifics share pests and pathogens, but also resource requirements, and therefore experience intense competition for obtaining resources, which contributes to mortality^8^.

Most of the results are inconsistent with most previous studies in both tropical^6^ and temperate forests^28^ that have found negative effects of con-specific neighbors on tree performance. These former studies tended to support the idea of negative density- and frequency-dependent effects. However, the results of our study indicate that there are positive effects of neighbors on survivorship of most of the species studied. The reason may be due to the complexity of habitat^43^ and spatial distributions of tree species of our 20-ha plot. In a former study about spatial patterns of three canopy species in our plot suggested that aggregated distribution was the dominant pattern in our plot, and aggregation was weaker in larger diameter classes^45^, especially spatial patterns and inter-specific associations of three canopy species (*Castanopsis chinensis, Schima superba, and Engelhardtia roxburghiana*) at different life stages in this plot^45^.

Most conceptual models of temperate old-growth forest dynamics assume that change is primarily driven by small scale disturbance such as wind, insects, and pathogens, and that competitive density-dependent mortality has ceased to play a major role, with the remaining large trees widely spaced and permissive of understory regeneration^30^. In contrast, He and Duncan^25^ inferred density-dependent mortality in old-growth conifer forests, but their studies were based on pattern analysis of a single census. Studies from mature and old-growth *Pinusresinosa* (red pine) forests in northern Minnesota found no evidence for density-dependent mortality^46^.

Traditionally, species within the same shade-tolerance class were expected to have similar responses to intrinsic and extrinsic factors. However, our results did not meet this expectation. Among the four shade-tolerant species the relative importance of the variables described above on tree species mortality varied significantly. There were no consistently positive or negative effects on tree survival for these four species. This contradiction between the shade-tolerance classes and species-level analysis may result from the fact that species within a particular shade tolerance classes often varied in terms of other characteristics that influenced survival patterns. In addition, tree size distribution varied greatly among these species^47^. These results are consistent with a recent study of seedling survival in a tropical forest where considerable variation occurred among individual species even within the same shade tolerance class^28^.

Demographic patterns and processes drive much of the spatial and temporal variation observed in forests. Mortality can create canopy gaps and microhabitats for tree recruitment that leave legacies in forest composition and structure^32^. Tree recruitment was influenced by multiple factors. The statistics of neighbors of recruited individuals (target tree) were used to conduct a comprehensive analysis of recruitment spatial pattern during the 5-year period. Our results indicated that tree recruitment was significantly influenced by neighboring trees. In general, the neighbors around the target tree had higher T-BA, Cons-BA, and Cons-N. But the recruits of almost all species studied had the same trend of observed results as randomly generated points at all four neighborhood radii.

Seed dispersal, seedling establishment, and sapling growth before tree recruitment (reach the DBH > 1 cm) may influence recruitment, and cause a bottleneck^8^. The variability in recruitment patterns suggests that both dispersal limitation and density dependence influenced recruitment^28^. In this study, the neighbors around the target tree had higher basal area and higher abundance of dead wood. In addition, some recruits tended to growth in forest gaps caused by tree mortality, others were shade-tolerant species. Species with different life-history strategies could also be influenced by intrinsic and extrinsic factors, according to many studies. Shade tolerance plays a central role in determining patterns of tree growth and survival^48^. Shade-tolerant species are less sensitive to shading by their neighbors, and they are less susceptible to enemy attacks than light-demanding species, based on differences in the allocation of resources to defense versus growth^13^. So the shade tolerance of trees should be a key trait in determining trees’ reactions to their local biotic neighborhood.

Though plants all consume a set of similar resources such as light, water and soil nutrient, different species may differ in the amount they require, and when they need it. Adjacent hetero-specific plants can influence each other by facilitation and competition. When the positive effect of facilitation exceeds that of competition, the net direction of plant-plant interaction is positive, and vice versa.

Using a 12-ha spatially explicit plot censused 13 years apart in an approximately 500-year-old *Pseudotsuga-Tsuga* forest, James A. Lutz *et al*.^49^ demonstrated significant density-dependent mortality and spatially aggregated tree recruitment. The combined effect of these strongly nonrandom demographic processes was to maintain tree patterns in a state of dynamic equilibrium. Overall changes in the abundance of species generally followed our expectations. Our results also indicate that although nearly all of the species in the DHS plot exhibited a stable population, the two most dominant species showed a declining population resulting from a lack of recruitment. The decline of two most dominant species matched predictions for this forest type: a gradual loss of the shade-intolerant pioneer cohort. One possible cause for this poor regeneration is that the relatively mature forest cannot meet the ecological needs of these species. *Castanopsis chinensis* and *S. superb* are considered to be moderately light-demanding or shade-intolerant species^50^. Similarly, reduction in recruitment by dominant species has been recorded in an African wet forest. Another possible explanation for poor regeneration in *Castanopsis chinensis* related to the biology of its seeds. Before dispersal, the seeds are predated by *Curuliodavidi*, a weevil, and then after dispersal by rats and birds^8^. Furthermore, pathogens threaten the survival of seeds in both pre- and post-dispersal periods^51^. Alternatively, recruits of these canopy species may be occurring at some distance away from maternal trees where canopy gaps are more prevalent. If this was indeed the case, such recruits outside of our study plot would have been missed in our survey.

Although much work has been done monitoring tree mortality and recruitment in this region of China, more studies over a larger area and longer time span are needed to elucidate whether the forest is at equilibrium in the short term. Also, to determine what factors are driving the succession process in this sub-tropical forest and other forests around the world.

## Conclusion

In summary, Dinghushan plot appears more dynamic than other forest plots based on comparisons of their demographic rates, but more studies are required to understand the mechanisms behind differences in vital rates. This study suggests that tree size and biotic factors contribute to the regulation of the DHS sub-tropical forest community, but that the relative importance of these factors differs among species. In addition, we found evidence that negative frequency dependence may not play a central role in the maintenance of diversity in this forest. Canopy species had difficulty recruiting into the ≥1-cm size class during the 5-year period and tree recruitment was related to shade tolerance. Tree mortality in this plot and recruitment for sixteen species was possibly limited by seed dispersal and density-dependence. Finally, we found that tree mortality was much more frequently associated with intra-specific competitive exclusion and density dependence than inter-specific interactions. Overall, the intra-specific effects observed in these forests appear to contribute to species coexistence. Therefore, the policy of forest community conservation and management should bear all of these variables in mind based on this study.

## Materials and Methods

### Study site

Our study site is located in the Dinghushan Nature Reserve (112°30′39″–112°33′41″E, 23°09′21″–23°11′30″N) in Guangdong Province, China^52^. The Dinghushan Nature Reserve was the first nature reserve to be established in China in 1956. The total area of the reserve is 1155 ha, with altitude varying from 14-1000 m, most of it covered by tropical-subtropical forests growing on lateritic red soil^43^. This region is characterized by a south subtropical monsoon climate. The mean annual temperature and precipitation are 20.9 °C and 1927 mm, respectively, and mean relative humidity is 85%.

### Field methods and biotic factors

We established a 20-ha permanent plot called the Dinghushan plot (DHS plot) in the Dinghushan Nature Reserve in 2004. The altitude of the plot ranges from 230 to 470 m, and the landform is highly complex, with steep slopes. The DHS plot (400 m × 500 m) was further divided into 500 subplots of 20 m × 20 m, and then into 8000 continuous 5 m × 5 m quadrats for the tree census. All stems within the 20-ha plot with a diameter at breast height (DBH) > = 1 cm were measured, mapped, and tagged when the plot was established in 2004–2005^43^,^53^. The second census was carried out between August and December in 2010. The DHS plot contained a total of 178 species in the second census in 2010. The status of trees (live or dead) was recorded in the second census^31^,^41^.

For tree mortality, we performed species-level analyses, by separately analyzing each of the 16 species with >1400 stems in 2005. In order to quantify the biotic neighborhood at the local scale, we use the proportion of con-specific neighbors (number of con-specific stems/number of all stems (Cons-N/T-N), basal area of con-specific stems/total basal area of all stems (Cons-BA/T-BA)), number of all stems (T-N), and the total basal area (T-BA) of the center of each focal tree. We chose 20 m because tree species interactions have been shown to disappear beyond 20 m^28^.

To determine whether recruitment was related to density, Cons-N, Cons-BA, T-N and T-BA within 5, 10, 15 and 20 m of recruits of the 16 dominant species, with the most recruits during the 5-year period between 2005 and 2010, were compared with those of 500 randomly generated points. If their 95% confidence intervals did not overlap they were considered to be significantly different. Here, recruitment means that the stems just reached the measure standard (DBH > = 1 cm) in the second census in 2010.

### Data analysis

We calculated demographic rates from the 5-year period census interval.


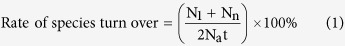


where N_1_ is the number of species lost from the census, N_n_ is number of new species appearing since the last census, and t is the time interval between the first and last census;


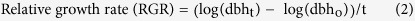


where dbh_o_ and dbh_t_ are the stem diameter at breast height (dbh) from first and last measurements, respectively;


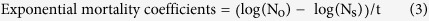


where N_o_ and N_s_ are the number of stems at the first measurement and number of surviving stems at the last measurement, respectively^3^;


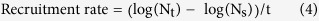


where N_t_ is number of stems at the last measurement;


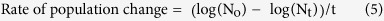


Generalized linear model (GLM) was used to model the probability of an individual tree survival in the 5-year census as a function of initial tree size in the first census (i.e., DBH) and the biotic neighborhood factors^54^. The GLM in this paper was essentially a logistic regression, with the response variables as tree status: 1 (alive) or 0 (dead)^47^. The resulting model was:


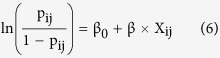


where p_ij_ is the probability of survival of trees; β_0_ is an intercept, β was a vector of model coefficients; i and j representing species and trees, respectively.

In this model, all the values of tree size (DBH) were log-transformed^11^. For all the explanatory variables (tree size and biotic factors), values were standardized by subtracting the mean value of the variables (across all individuals in the analysis) and dividing by standardized deviation. This allows for a direct comparison of the relative importance of these explanatory variables^47^. To avoid edge effects, we also excluded all potential target trees that were within 20 m of the edge of the plot from the analyses. As measures of model predictive and discriminative ability, Nagelkerke’s R^2^_N_ was used to detect the accuracy of the best–fit models^55^. All the calculations were carried out in R version 3.1.3 Team^56^.

## Additional Information

**How to cite this article**: Ma, L. *et al*. Forest dynamics and its driving forces of sub-tropical forest in South China. *Sci. Rep.* **6**, 22561; doi: 10.1038/srep22561 (2016).

## Figures and Tables

**Table 1:** Difference in the number of individuals, number of dead wood and recruits for the 20 species that reduced most during the 5-year in DHS plot.

**Species**	**N2005**	**N2010**	**Variation**	**Dead**	**Recruits**
*Craibiodendron scleranthum* var. * Kwangtungense* (S. Y. Hu) Judd	3287	1784	−1503	1513	10
*Lindera metcalfiana* Allen	2113	924	−1189	1293	104
*Blastus cochinchinensis* Lour	3994	2944	−1050	1309	259
*Aporosa yunnanensis* Metc	3722	2789	−933	981	48
*Lindera chunii* Merr	1293	409	−884	897	13
*Neolitsea pallens* (D. Don) Momiyama et Hara	1326	474	−852	863	11
*Syzygium rehderianum* Merr. et Perry	5917	5103	−814	899	85
*Cryptocarya concinna* Hance	4449	3829	−620	1595	975
*Ardisia quinquegona* Bl	3696	3127	−569	651	82
*Garcinia oblongifolia* Champ	612	77	−535	540	5
*Psychotria asistica* Linn.	902	455	−447	462	15
*Ormosia glaberrima* Wu	2752	2344	−408	511	103
*Schima superba* Gardn. et Champ	2290	1894	−396	406	10
*Rhododendron henryi* Hance	1404	1103	−301	310	9
*Cryptocarya chinensis* (Hance) Hemsl	2546	2268	−278	375	97
*Sarcosperma laurinum* (Benth.) Hook. f	1578	1351	−227	271	44
*Castanopsis chinensis* Hance	2294	2076	−218	231	13
*Canthium dicoccum* (Gaertn.) Merr	605	395	−210	248	38
*Machilus breviflora (Benth.)* Hemsl	844	636	−208	214	6
*Acmena acuminatissima* Merr. et Perry	1526	1377	−149	199	50

**Table 2:** Difference in the number of individuals, number of dead wood and recruits for the 20 species that increased most during the 5-year in DHS plot.

**Species**	**N2005**	**N2010**	**Variation**	**Dead**	**Recruits**
*Aidia canthioides* (Champ. ex Benth.) Masamune	5969	7894	1925	479	2404
*Carallia brachiata* (Lour.) Merr.	747	985	238	72	310
*Castanopsis fissa* (Champ.) R. et W.	263	480	217	73	290
*Cryptocarya chinensis* (Hance) Hemsl.	155	309	154	43	197
*Elaeocarpus dubius* A. DC.	344	459	115	59	174
*Pygeum topengii* Merr	114	139	25	22	47
*Canarium album* Raeuch	375	399	24	67	91
*Ilex ficoidea* Hemsl	617	633	16	21	37
*Nageia fleuryi (Hickel) de Laub.*	5	18	13	0	13
*Caryota maxima* Blune	38	48	10	5	15
*Sterculia lanceolata* Cav	52	60	8	6	14
*Bridelia fordii* Hemsl.	80	88	8	16	24
*Canthium horridium* Bl.	25	31	6	3	9
*Laurocerasus phaeosticta* (Hance) S. K. Schneid	28	34	6	3	9
*Neolitsea chuii* Merr	7	11	4	0	4
*Wikstroemia nutans* Champ. Ex Benth	19	23	4	5	9
*Bridelia tomentosa* Bl. [B. monoica (Linn.) Merr.]	0	3	3	0	3
*Trema angustifolia* Bl.	3	5	2	3	5
*Glycosmis parviflora* (Sims) Little	4	6	2	0	2
*Clerodendrum fortunatum* L.	2	4	2	2	4

**Table 3:** Summary of generalized linear model analyses of 16 species with >1400 individuals in the DHS plot.

**Species**	**Log(dbh)**	**T-BA**	**T-N**	**Cons-BA**	**Cons-N**	**R**^**2**^_**N**_	**Dispersal modes**	**Shade tolerance**
*Aidia canthioides* (Champ. ex Benth.) Masamune.	0.18	0.32***	0.18	−1.48	0.33***	0.026	Wind	mid-tolerant
*Syzygium rehderianum* Merr. et Perry	0.35***	0.13*	0.45***	−0.41*		0.026	Wind	mid-tolerant
*Blastus cochinchinensis* Lour	−0.64***	0.11*	−0.08	0.11	−0.03	0.025	Wind	shade-tolerant
*Cryptocarya concinna* Hance	−0.34***	0.02	0.17**	0.23	0.22***	0.027	Animal	mid-tolerant
*Ardisia quinquegona* Bl	0.38**	0.12*	0.36***	−0.24	0.05	0.021	Wind	mid-tolerant
*Aporosa yunnanensis* Metc	0.38***	0.18**	0.19*	0.37	0.08	0.046	Animal	shade-tolerant
*Cryptocarya chinensis* (Hance) Hemsl	0.10	0.05	−0.01	0.09	−0.34**	0.030	Animal	mid-tolerant
*Craibiodendron scleranthum var. kwangtungense* (S. Y. Hu) Judd	0.91***	−0.11	0.16*	0.24*	−0.14	0.128	Animal	mid-tolerant
*Ormosia glaberrima* Wu	−0.17**	0.16	0.21*	0.11	−0.10	0.012	Animal	mid-tolerant
*Castanopsis chinensis* Hance	0.74***	−0.15	0.09	0.10	−0.13	0.049	Animal	mid-tolerant
*Schima superba* Gardn. et Champ	1.91***	−0.17	0.87***	−0.17	0.69**	0.093	Animal	mid-tolerant
*Lindera metcalfiana* Allen	−0.20**	−0.20**	0.18*	0.42	0.13	0.038	Animal	mid-tolerant
*Lindera chunii* Merr	−0.25**	−0.04	0.03	−0.02	0.86***	0.039	Animal	mid-tolerant
*Xanthophyllum hainanense* Hu	0.80***	0.21	−0.06	0.30	0.36	0.035	Animal	mid-tolerant
*Acmena acuminatissima* Merr. et Perry	0.25***	0.14	0.19	0.05	−0.59*	0.025	Animal	shade-tolerant
*Sarcosperma laurinum* (Benth.) Hook. f	0.43***	0.11	0.19	0.03	−0.11	0.034	Wind	shade-tolerant

“T-BA” stands for total basal area of all stems; “T-N” stands for total number of all stems; “Cons-BA” stands for basal area of con-specific stems; “Cons-N” stands for number of con-specific stems.

**Table 4:** Summary of results for the recruitment’s analysis (with live woods).

**Species**	**T-BA**	**Cons-BA**	**T-N**	**Cons-N**	**Scale**
*Aidia canthioides* (Champ. ex Benth.) Masamune.	↑	−	↓	↑	N
*Cryptocarya concinna* Hance	↑	↑	↓, −	↑	Y
*Blastus cochinchinensis* Lour	↓, −	↑	↑, ↓, −	↑, ↓, −	Y
*Carallia brachiata* (Lour.) Merr.	↑, −	↑	↓	↑	Y
*Castanopsis fissa* (Champ.) R. et W.	↓, −	↑	↑	↑	Y
*Cryptocarya chinensis* (Hance) Hemsl	↑, −	↑	↓	↑	Y
*Cryptocarya concinna* Hance	↑, −	↑, −	−	↑	Y
*Ardisia quinquegona* Bl	↑, −	↑	↑	↑	Y
*Mallotus paniculatus* (Lam.) Muell. -Arg	↓, −	↑, −	−	↑	Y
*Lindera metcalfiana* Allen	↑	↑	↓	↑	N
*Castanopsis chinensis* Hance	↑	↑	↓, −	↑	Y
*Elaeocarpus dubiu*s A. DC.	↑	↑, −	−	↑	Y
*Ormosia glaberrima* Wu	↓, −	↑, −	↑	↑	Y
*Acmena acuminatissima* Merr. et Perry	↓	↑	−	↑	N
*Engelhardtia roxburghiana* Wall	↑, −	↑	↑	↑	Y
*Schima superba* Gardn. et Champ	−	↑, −	−	↑, −	Y

“T-BA” stands for total basal area of all stems; “T-N” stands for total number of all stems; “Cons-BA” stands for basal area of con-specific stems; “Cons-N” stands for number of con-specific stems. “↑” stands for values of observation significantly higher than values of simulation; “↓” stands for values of observation significantly lower than values of simulation; “−” stands for values of observation and simulation have no significantly difference; “Y” stands for differences between values of observation and simulation have the same trend at all four neighborhood radii; “N” stands for differences between values of observation and simulation have the different trend at all four neighborhood radii.

**Table 5:** Summary of results for the recruitment’s analysis (with dead woods).

**species**	**T-BA**	**T-N**	**Scale**
*Aidia canthioides* (Champ. ex Benth.) Masamune.	↑, −	↓	Y
*Cryptocarya concinna* Hance	↑	↑, −	Y
*Blastus cochinchinensis* Lour	↓, −	↑, −	Y
*Carallia brachiata* (Lour.) Merr.	↓, −	↓	Y
*Castanopsis fissa* (Champ.) R. et W.	↑, −	↑	Y
*Cryptocarya chinensis* (Hance) Hemsl	−	↓	N
*Cryptocarya concinna* Hance	−	−	N
*Ardisia quinquegona* Bl	↓, −	↑	Y
*Mallotus paniculatus* (Lam.) Muell. -Arg	↑	↑	N
*Lindera metcalfiana* Allen	↑	↑, −	Y
*Castanopsis chinensis* Hance	−	↓, −	Y
*Elaeocarpus dubiu*s A. DC.	↑, −	↑, −	Y
*Ormosia glaberrima* Wu	↓	↑, −	Y
*Acmena acuminatissima* Merr. et Perry	−	↓, −	Y
*Engelhardtia roxburghiana* Wall	↑, −	↑	Y
*Schima superba* Gardn. et Champ	↑, −	↑, −	Y

“T-BA” stands for total basal area of all stems; “T-N” stands for total number of all stems; “Cons-BA” stands for basal area of con-specific stems; “Cons-N” stands for number of con-specific stems. “↑” stands for values of observation significantly higher than values of simulation; “↓” stands for values of observation significantly lower than values of simulation; “−” stands for values of observation and simulation have no significantly difference; “Y” stands for differences between values of observation and simulation have the same trend at all four neighborhood radii; “N” stands for differences between values of observation and simulation have the different trend at all four neighborhood radii.
